# Layer-specific high-frequency action potential spiking in the prefrontal cortex of awake rats

**DOI:** 10.3389/fncel.2013.00099

**Published:** 2013-06-26

**Authors:** Zimbo S. R. M. Boudewijns, Martine R. Groen, Brendan Lodder, Minni T. B. McMaster, Lawrence Kalogreades, Roel de Haan, Rajeevan T. Narayanan, Rhiannon M. Meredith, Huibert D. Mansvelder, Christiaan P. J. de Kock

**Affiliations:** Department of Integrative Neurophysiology, Centre for Neurogenomics and Cognitive Research, Neuroscience Campus Amsterdam, Vrije Universiteit AmsterdamAmsterdam, Netherlands

**Keywords:** calcium electrogenesis, prefrontal cortex, action potential, high-frequency bursts, dendrites, backpropagation, *in vivo*, awake rats

## Abstract

Cortical pyramidal neurons show irregular *in vivo* action potential (AP) spiking with high-frequency bursts occurring on sparse background activity. Somatic APs can backpropagate from soma into basal and apical dendrites and locally generate dendritic calcium spikes. The critical AP frequency for generation of such dendritic calcium spikes can be very different depending on cell type or brain area involved. Previously, it was shown *in vitro* that calcium electrogenesis can be induced in L(ayer) 5 pyramidal neurons of prefrontal cortex (PFC). It remains an open question whether somatic burst spiking and the resulting dendritic calcium electrogenesis also occur in morphologically more compact L2/3 pyramidal neurons. Furthermore, it is not known whether critical frequencies that trigger dendritic calcium electrogenesis occur in PFC under awake conditions *in vivo*. Here, we addressed these issues and found that pyramidal neurons in both PFC L2/3 and L5 in awake rats spike APs in short bursts but with different probabilities. The critical frequency (CF) for calcium electrogenesis *in vitro* was layer-specific and lower in L5 neurons compared to L2/3. Taking the *in vitro* CF as a predictive measure for dendritic electrogenesis during *in vivo* spontaneous activity, supracritical bursts *in vivo* were observed in a larger fraction of L5 neurons compared to L2/3 neurons but with similar incidence within these subpopulations. Together, these results show that in PFC of awake rats, AP spiking occurs at frequencies that are relevant for dendritic calcium electrogenesis and suggest that in awake rat PFC, dendritic calcium electrogenesis may be involved in neuronal computation.

## Introduction

Sparse spiking activity in combination with short bursts of two or three spikes at high frequency is a characteristic feature of cortical areas and has been documented during anesthesia as well as wakefulness (Barth and Poulet, 2012). Sparse spiking optimizes information content (Olshausen and Field, 2004), whereas bursts increase the reliability of synaptic transmission through facilitation of neurotransmitter release (Lisman, 1997). Action potentials (APs) can additionally backpropagate into distal dendritic compartments to increase computational power at the cellular level (Larkum, 2013). Backpropagating single APs typically attenuate but AP bursts above a critical frequency (CF) can induce calcium-mediated spikes in distal dendritic compartments (Larkum et al., 1999a). Calcium-mediated dendritic spikes are particularly well studied in cortical L5B thick tufted neurons and can be the outcome of either a combination of a backpropagating single AP coinciding with distal synaptic input (Larkum et al., 1999b) or occur through somatic AP bursts exceeding the CF for dendritic electrogenesis (Larkum et al., 1999a). In turn, dendritic spikes are involved in plasticity mechanisms (Kampa et al., 2006; Nevian and Sakmann, 2006) and associative mechanisms to integrate segregated information streams in cortex (Larkum, 2013). Together, these findings indicate that bursts and the generation of dendritic calcium events serve a critical physiological role in cortical networks (Larkum et al., 2009).

Burst spiking-induced calcium electrogenesis occurs in a variety of cell types in somatosensory cortex (Williams and Stuart, 1999; Nevian and Sakmann, 2004; Perez-Garci et al., 2006; Ledergerber and Larkum, 2010), entorhinal cortex (Medinilla et al., 2013), hippocampus (Takahashi and Magee, 2009), and prefrontal cortex (PFC) (Seamans et al., 1997; Gulledge and Stuart, 2003; Barth et al., 2008). However, the cell-type-specific characteristics of AP backpropagation and associated calcium electrogenesis in the PFC remain largely unknown. Moreover, it is not known whether *in vivo* AP spiking occurs at frequencies sufficient for calcium electrogenesis in the PFC.

Here, we investigated the cell-type-specific AP spiking activity and the occurrence of burst spiking in rat PFC. We found sparse spiking activity in both L2/3 and L5 pyramidal neurons of PFC in awake rats but also high-frequency bursts. We then examined the frequency-dependent increase in afterdepolarization (ADP) *in vitro*, which was previously shown to be a reliable readout for the frequency-dependent induction of dendritic calcium spikes (Larkum et al., 1999a; Perez-Garci et al., 2006; Potez and Larkum, 2008; Ledergerber and Larkum, 2010). We found that the CF for the change in ADP amplitude was layer-specific and we found that L2/3 and L5 neurons in the PFC of awake rats regularly spike at frequencies above the CF obtained *in vitro*, suggesting potential dendritic electrogenesis *in vivo*. Finally, the occurrence of supracritical bursts *in vivo* was layer-specific and supracritical bursts were observed in a larger fraction of L5 neurons compared to L2/3 neurons.

## Materials and methods

### Animal preparation

All experiments were carried out in accordance with the animal welfare guidelines of the Vrije Universiteit Amsterdam, The Netherlands. For *in vivo* experiments, male and female Wistar rats [Harlan, Charles River, postnatal day (PND) 27–44, 116.4 ± 24.3 g] were used. To avoid effects of stress and prolonged exposure to anesthetics associated with the surgery, rats underwent surgery one day prior to the recording session. Rats were anesthetized using 1.75% isoflurane in 0.4 l/h O_2_ + 0.7 l/h NO_2_ and depth of anesthesia was assured by absence of foot and eyelid reflexes. Animal body temperature was monitored using a rectal probe and maintained at 36–37°C with a heating pad. Rats were positioned in the recording setup using a head post. To investigate possible effects of novelty-induced stress from head fixation on neuronal spiking activity, we habituated a subset of animals to the recording procedure (head fixation two times per day for 2–3 days before the recording session). No effect of habituation on spiking frequencies was observed for either L2/3 neurons [habituated: 0.17(0.25), *n* = 7; non-habituated: 0.11(0.38), *n* = 9, Mann–Whitney U test, *p* = 0.67] or L5 neurons [habituated: 0.25(0.92), *n* = 10; non-habituated: 1.07(0.47), *n* = 6, *p* = 0.23]. On the recording day, rats were anesthetized with isoflurane (1.25% in 0.4 l/h O_2_ + 0.7 l/h NO_2_) to obtain stable juxtasomal recordings after which anesthesia was terminated. In a similar setting designed to study EEG signals during transition from isoflurane anesthesia to awake conditions, spontaneous body movements by the animal were used to define the moment of awakening (Kortelainen et al., 2012). In our experiments, UP and DOWN states in the local field potential disappeared almost instantaneously upon termination of isoflurane anesthesia and muscle tone increased after 2–3 min, followed by spontaneous large-amplitude exploratory whisking. In all our experiments, we waited until animals displayed exploratory, large-amplitude whisking, which was often accompanied by full body movements, including hind- and forepaw movements. Awake recordings used for analysis were obtained 2–30 min after first exploratory whisking was observed.

Spiking frequencies during urethane (intraperitoneally, 1.6–1.7 g kg^−1^) and isoflurane anesthesia (1.25%, in 0.4 l/h O_2_ + 0.7 l/h NO_2_) were also obtained (data reported in text).

### *In vivo* electrophysiology

Juxtasomal recordings were made as previously described (de Kock et al., 2007). Individual neurons were recorded using patch electrodes (5–7 MΩ) filled with (in mM): 135 NaCl, 5.4 KCl, 1.8 CaCl_2_, 1 MgCl_2_ and 5 Hepes, 2% biocytin, pH adjusted to 7.2 with NaOH. Electrodes were positioned 300 (L2/3) or 700 (L5) μm lateral to the midline of the left PFC (rostrocaudal: 3 mm, dorsoventral 2–3 mm) to target the prelimbic part of PFC. To ensure unbiased sampling (irrespective of spiking frequency), single neurons were searched for by monitoring electrode resistance while lowering the electrode in 1 μm steps. Spontaneous activity was monitored for 50–125 s after which neurons were filled with biocytin using electroporation (Pinault, 1996; Joshi and Hawken, 2006).

### *In vitro* electrophysiology

For *in vitro* experiments, male Wistar rats (PND 33–37) were used. Rats were decapitated after which their brains were transferred into ice cold slice solution containing (in mM): 110 choline chloride, 11.6 Na-ascorbate, 3.1 Na-pyruvate, 2.5 KCl, 1.25 NaH_2_PO_4_, 7 MgCl_2_, 0.5 CaCl_2_, 10 glucose, and 26 NaHCO_3_. 350 μm PFC slices were cut using a LEICA VT1000S vibratome. Slices were stored at 35°C for 20 min in artificial cerebrospinal fluid (aCSF) containing (in mM): 125 NaCl, 3 KCl, 1.2 NaH_2_PO_4_, 1 Mg SO_4_, 2 CaCl_2_, 10 glucose, and 26 NaHCO_3_ and stored at room temperature until the start of the recording. All recordings were made in aCSF at 32°C.

Whole cell patch-clamp recordings were made from L2/3 and L5 PFC neurons using 2.5–4.5 MΩ pipettes filled with intracellular solution containing (in mM): 135 K-gluconate, 7 KCl, 10 HEPES, 4 Mg-ATP, 10 Na_2_ phosphocreatine, 0.3 GTP, and 0.2% biocytin. Recordings were excluded when series resistance exceeded 20 MΩ or changed more than 20% during the recording. Neurons without *post-hoc* identification or without full apical dendritic morphology were excluded from analysis.

Passive membrane properties were determined based on 500 ms hyperpolarizing and depolarizing current steps injected at the soma, starting at −100 pA and increased with steps of 25 pA. Input resistance was determined by a linear fit on the IV curve excluding steps that resulted in AP spiking. The current mediated by hyperpolarization-activated cation channels (*I*_h_) was assessed by measuring the difference between the maximum deflection and the steady state deflection after a −100 pA hyperpolarizing current step. Junction potential was not corrected for during recordings.

Two or three APs with varying frequencies (5–160 Hz) were evoked in a pseudorandom order by applying 2 ms electrical pulses. First, we determined the frequency-dependent amplitude of the ADP in a 10–30 ms window after the last AP, since frequency-dependent changes in ADP amplitude are indicative of dendritic electrogenesis (Larkum et al., 1999a; Perez-Garci et al., 2006; Potez and Larkum, 2008; Ledergerber and Larkum, 2010). Second, the presence of a CF was determined by fitting the sigmoidal curve (Figure 3G):

$$y(f)=B+A\;\cdot \;\frac{1}{1+e^{-s\;\cdot \;(f\;-\;CF)}}$$

where *f* represents the AP frequency, *B* is baseline ADP, *A* represents the amplitude of the frequency-dependent ADP change, *s* is the slope of the fit, and *CF* is the critical frequency. Neurons were defined to have a significant CF when the 95% confidence interval of the sigmoidal curve fit excluded zero. When the slope of the frequency–ADP relationship could not be fitted by a sigmoidal curve, we concluded that dendritic electrogenesis could not be evoked. For these neurons, the size of the ADP was thus frequency-independent. As an alternative method, we determined the ADP area in the same window (10–30 ms) and used the ADP-area values to fit the sigmoidal curve. This analysis resulted in statistically similar CF values (Spearman *R*^2^ = 0.71, Wilcoxon signed rank, *p* = 0.14, data not shown).

### *In vitro* and *in vivo* histology

After *in vivo* experiments, rats were perfused with 0.9% NaCl, then 4% paraformaldehyde [PFA, in 0.11 M phosphate buffer (PB)]. Brains were post-fixed in 4% PFA for 1–3 weeks at 4°C. Coronal sections (100 μm) were cut on a vibratome and stained using a modified avidin–biotin–peroxidase method (Horikawa and Armstrong, 1988). To enhance biocytin signals, avidin–biotin complex (ABC) incubated slices were reacted for 5 min in 0.45 μg/ml tyramine (Sigma-Aldrich) and 0.875 μg/ml biotin NHS dissolved in PB, then 15 min in the same tyramine solution with 0.003% H_2_O_2_ added (Furuta et al., 2011). Slices were then re-incubated in ABC for 2–3 h and stained using 3,3′-diaminobenzidine. *In vitro* slices were stained following conventional DAB staining.

Typically, recorded neurons showed sufficient biocytin label to determine the layer location, but in a small fraction, juxtasomal biocytin labeling failed. For these neurons, adjacent recorded and labeled neurons or electrode tracks were used for layer classification. Recordings for which layer location could not be identified were discarded. A subset of neurons showed sufficient biocytin signal to allow reconstructions of the dendritic morphology. These neurons were reconstructed using Neurolucida software (Microbrightfield, Williston, VT, USA) using a 100× oil immersion objective (Olympus, N.A. 1.4). Neurons were classified as L2/3 or L5 neurons at 10× magnification using a bright field microscope (CX31, Olympus). When soma location was near the L3–L5 border, slices contralateral to the recovered neuron were Nissl stained and overlaid with the DAB stained section to determine layer location. Using Nissl staining, the L3–L5 border can be readily discriminated by a sharp increase in cell body density in L5 (Van Eden and Uylings, 1985). For Nissl staining, slices were rinsed in Na acetate buffer (0.5442% NaOAc and 0.9608% acetic acid) and incubated for 10–15 min in 0.5% Cresyl violet. Immediately after Cresyl violet staining, slices were coverslipped and images were taken at 4× magnification.

## Data analysis

Electrophysiological data was almost exclusively non-parametrically distributed and population data is therefore reported as median and interquartile range (IQR, between brackets) unless stated otherwise. Statistical analyses were performed using Graphpad InStat 3 (GraphPad Software, Inc., La Jolla, USA) and Matlab R2009b (Mathworks, Natick, USA).

## Results

**Figure 1:**
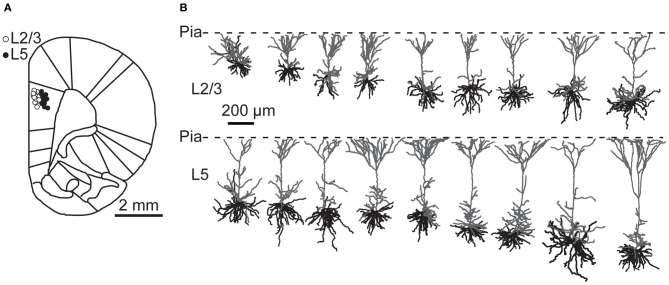
**Recording locations and dendritic morphology of prelimbic PFC pyramidal neurons. (A)** Cartoon of a coronal section through rat prefrontal cortex with superimposed recording locations of prelimbic PFC neurons. Open circles depict L2/3 neurons; filled circles L5 neurons. **(B)** Reconstructions of L2/3 and L5 neurons from the PFC from anesthetized and awake animals. Apical dendrites are shown in gray and basal dendrites in black.

To examine ongoing and instantaneous spiking frequencies, PFC pyramidal neurons were recorded in anesthetized and awake rats. Recorded neurons were biocytin labeled and neurons were identified based on the location of their cell body with respect to the cytoarchitectonic layers (Figure 1A). Neurons with strong biocytin signal were 3D reconstructed and revealed typical pyramidal architecture with multiple basal dendrites and a single tufted apical dendrite projecting to the pia (Figure 1B). Previous studies have subclassified L5 PFC neurons into two subtypes on the basis of their axonal target areas and showed that differences in dendritic morphology also emerge when the projection target is used as the primary classification parameter (Morishima and Kawaguchi, 2006; Dembrow et al., 2010). However, our reconstructions are based only on dendritic morphology, which is not sufficient to reliably subclassify reconstructed neurons. In addition, only for a subset of recovered neurons, biocytin signal of the recorded neuron was sufficient for complete dendrite reconstruction. Consequently, we did not have a sufficient number of reconstructed neurons to subclassify neurons on the basis of dendritic morphology and we pooled data on spiking activities obtained from L5.

**Figure 2:**
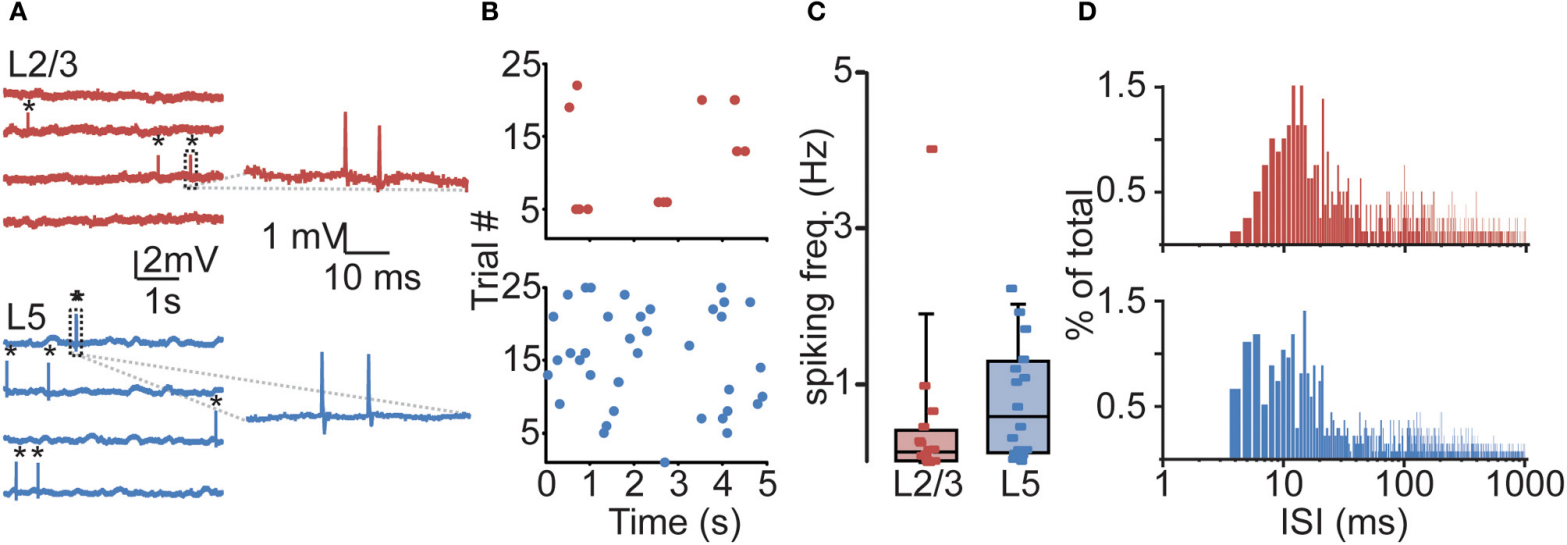
**Sparse and irregular action potential (AP) spiking activity in prelimbic PFC of awake rats. (A)** Representative juxtasomal recordings of individual L2/3 and L5 neurons recorded during wakefulness. APs are indicated by black asterisks. Right panel shows magnification of area indicated in dashed box. **(B)** Raster plots of the neurons shown in **(A)** showing all spikes observed during a 125 s recording. **(C)** Box plot showing cell-type-specific median spiking frequencies for L2/3 (*n* = 16) and L5 (*n* = 16) neurons in awake animals. No difference could be observed in spiking activity between L2/3 and L5 neurons. **(D)** Histogram illustrating the distribution of interspike intervals (ISIs) for L2/3 (upper panel) and L5 (lower panel). Note the positive skew in the distribution, indicating that APs are often found in close temporal proximity.

All neurons displayed sparse, irregular spiking activity with bursts intermingled with episodes of relative silence (Figures 2A,B). During recording episodes of 50–125 s, ongoing spiking frequencies for individual neurons were in the range of 0.01–4.03 Hz (L2/3) and 0.02–2.24 Hz (L5) and typically below 1 Hz. Median spiking rates in awake rats did not differ significantly between L2/3 and L5 (L2/3 0.14 Hz, L5 0.59 Hz, Figure 2C, Table 1, Kruskal–Wallis with Dunn's *post-hoc* test, *p* > 0.05).

**Table 1:** **Ongoing spiking frequencies (in Hz) during anesthetized and awake recordings**.

	**L2/3**		**L5**		**L2/3 vs. L5**
	**Median (IQR)**	***n***	**Median (IQR)**	***n***	
Awake	0.14 (0.29)	16	0.59 (1.08)	16	*p* > 0.05
Isoflurane	0.10 (0.24)	31	0.28 (0.34)	29	*p* < 0.05
Urethane	0.27 (0.68)	15	1.00 (2.37)	20	*p* < 0.05
Isoflurane-to-awake	0.08 (0.08)−0.98 (1.63)	4	0.48 (0.90)−1.03 (2.01)	6	

To determine spiking rates in PFC under anesthetized conditions, we recorded spiking frequencies during isoflurane (1.25%) and urethane anesthesia (Table 1). During isoflurane anesthesia, L5 neurons showed significantly higher spiking frequencies compared to L2/3 neurons (0.28 vs. 0.10 Hz, respectively, *p* < 0.05). Also during urethane anesthesia, spiking frequencies were higher in L5 compared to L2/3 (1.00 vs. 0.27 Hz, respectively, *p* < 0.05). For both L2/3 and L5 neurons, spiking frequencies were similar between awake, urethane, and isoflurane anesthesia (*p* > 0.05). Spiking frequencies quantified for neurons that were continuously recorded from the anesthetized (1.25% isoflurane, *n* = 10) to the awake state show that, in L5, spiking frequencies increased significantly from the anesthetized to the awake state (0.48 vs. 1.03 Hz, respectively; Wilcoxon signed rank test, *p* < 0.05), but no such effect could be observed for L2/3 neurons (0.08 vs. 0.98 Hz, respectively, *p* = 0.13). Thus, we observed subtle effects of isoflurane on spiking activity but only in L5 and when testing both conditions within individual neurons. In conclusion, L5 neurons of PFC show significantly higher ongoing spiking rates compared to L2/3 neurons but only in anesthetized (urethane or isoflurane) conditions. In PFC of awake rats, spontaneous ongoing activity is statistically comparable for L2/3 and L5 neurons.

The distribution of interspike intervals (ISIs) was not normally distributed for both L2/3 (Kolmogorov–Smirnov test, *p* < 0.001) and L5 (*p* < 0.001) and distributions for both L2/3 and L5 were positively skewed, indicating that APs were more often observed in close temporal proximity (Figure 2D, skewness L2/3: 6.63, L5: 10.18). Instantaneous spiking frequencies during bursts (calculated on the basis of ISIs) were typically in the order of 40–150 Hz. Since calcium electrogenesis in somatosensory cortex occurs during bursts when instantaneous frequencies exceed on average a CF of 100 Hz (Larkum et al., 1999a, 2007), spiking rates in PFC could suggest that AP frequencies during bursts also generate dendritic calcium spikes.

**Figure 3:**
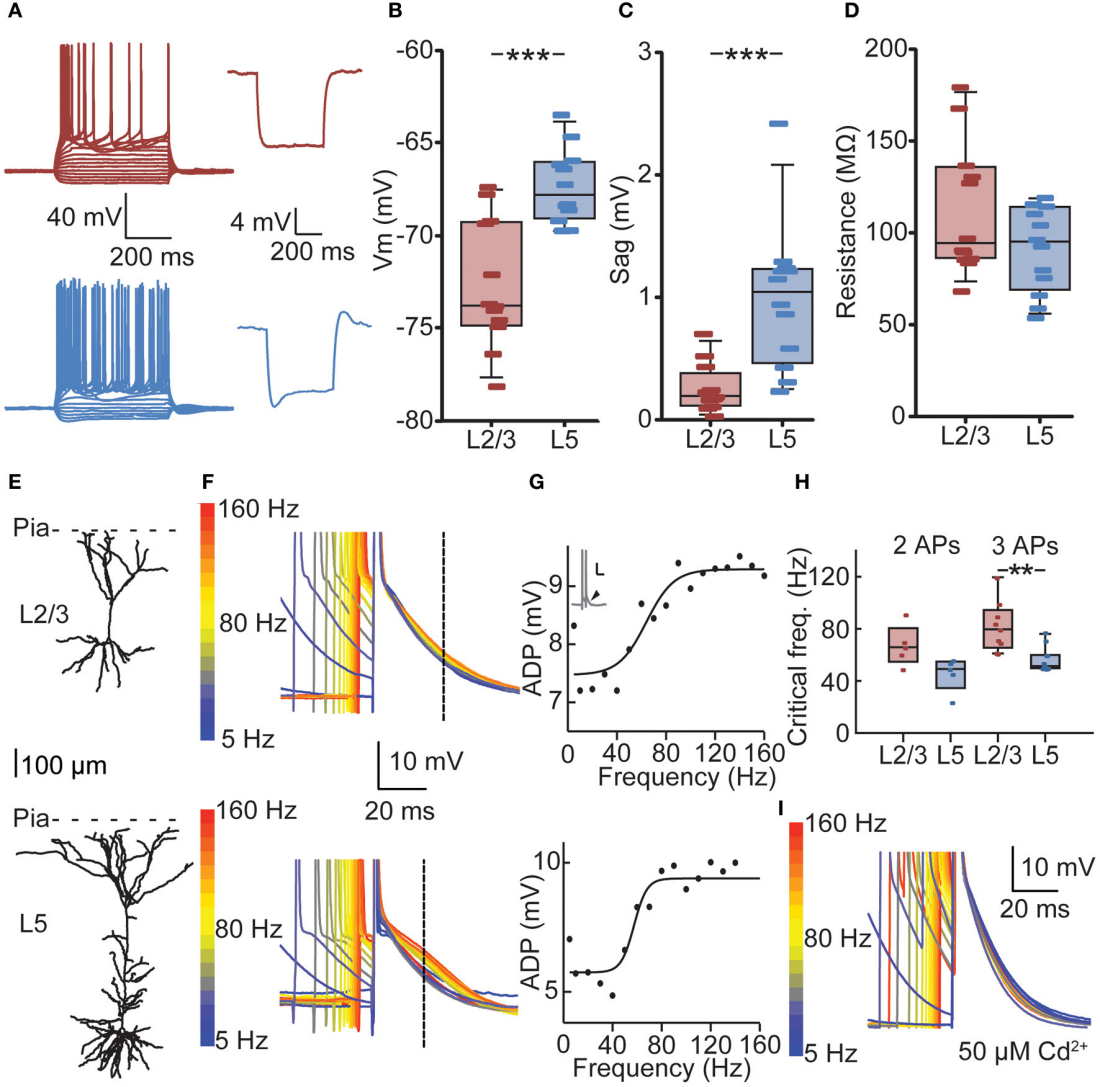
**Cell-type-specific critical frequency (CF) for dendritic calcium electrogenesis in PFC. (A)** Example traces of depolarizing and hyperpolarizing current steps in L2/3 (red) and L5 (blue) neuron (left panels) and traces showing hyperpolarization-activated cation current (*I*_h_) after 100 pA hyperpolarizing current step for a L2/3 (red) and L5 (blue) neuron. **(B)** L2/3 neurons showed a significantly lower resting membrane potential compared to L5 neurons. **(C)** L5 neurons show more prominent *I*_h_ current compared to L2/3 neurons, as indicated by a larger sag. **(D)** No difference could be observed in the input resistance (*R*_in_) of L2/3 and L5 neurons. **(E)** Example of a reconstructed L2/3 (upper panel) and L5 neuron (lower panel). **(F)** Voltage traces derived from the L2/3 neuron (upper panel) and the L5 neuron (lower panel) depicted in **(E)**. Two APs in a burst with varying ISIs are shown, aligned to the last AP in the sweep. Color coding denotes low frequency (blue) to high-frequency (red) bursts. Dashed line indicates the point at which the size of the ADP was determined. **(G)** Fitting of a sigmoidal curve to the ADP data reveals a non-linear increase in the ADP at CFs for both the L2/3 neuron (upper panel) and the L5 neuron (lower panel). **(H)** CFs did not differ whether two or three APs were evoked. The CF for L2/3 neurons was statistically comparable to CF for L5 neurons using two APs, but CF of L5 neurons was significantly lower when three APs were used. **(I)** The non-specific voltage-dependent calcium channel blocker cadmium (50 μm) abolished any frequency-dependent increase in ADP amplitude. ^**^denotes *p* < 0.05, ^***^denotes *p* < 0.01.

To determine whether bursts at frequencies observed *in vivo* meet the CF for dendritic calcium electrogenesis in PFC L2/3 and L5 neurons, we made current clamp recordings in acute PFC slices. We first measured passive and active membrane properties of L2/3 and L5 neurons (Figure 3A, Table 2) and found layer-specific differences in resting membrane potential (*V*_m_, Figure 3B) and sag amplitude (indicative of *I*_h_ expression, Figure 3C), but not input resistance (*R*_in_, Figure 3D). More specifically, L5 neurons were significantly more depolarized than L2/3 neurons (Figure 3B, −67.77 mV vs. −73.74 mV, respectively, Mann–Whitney U test, *p* < 0.001). In accordance with a previous study (van Brederode and Spain, 1995), we found a significantly lower *I*_h_ sag in L2/3 compared to L5 neurons (Figure 3C, 0.21 vs. 1.05 mV, respectively, Mann–Whitney U test, *p* < 0.001). No differences could be observed in the input resistance between the two layers (Figure 3D, L2/3: 94.15 MΩ, L5: 94.95 MΩ, Mann–Whitney U test, *p* = 0.20). In our data, passive and active properties of L2/3 and L5 neurons were distributed continuously, indicating two homogenous groups of neurons recorded in these experiments. On this basis, CF measurements were pooled for each layer.

**Table 2:** **Passive properties of *in vitro* recorded L2/3 (*n* = 12) and L5 (*n* = 12) neurons**.

	**L2/3**	**L5**	**L2/3 vs. L5**
	**Median (IQR)**	**Median (IQR)**	
*V*_*m*_ (mV)	−73.74 (5.33)	−67.77 (2.64)	*p* < 0.001
*I*_*h*_ sag (mV)	0.21 (0.14)	1.05 (0.68)	*p* < 0.001
*R*_in_ (MΩ)	94.15 (46.29)	94.95 (38.26)	*p* = 0.20

We determined the CF for dendritic calcium electrogenesis by evoking two or three somatic APs at different frequencies. Above the CF, these somatic APs induce calcium electrogenesis, which can be measured as a frequency-dependent (sigmoidal) increase in the AP ADP (see Materials and Methods) (Larkum et al., 1999a; Perez-Garci et al., 2006; Potez and Larkum, 2008; Ledergerber and Larkum, 2010). In both L2/3 and L5 pyramids, we observed a supralinear increase in ADP amplitude at higher AP frequencies (Figures 3E–G). With two APs, the average CF for an increase in ADP in L2/3 was 67 Hz, but the sigmoidal curve could only be adequately fitted in 5 out of 12 recordings, indicating that in 7 out of 12 neurons no increase in ADP was observed, irrespective of the evoked AP frequency (see Materials and Methods, Figure 3G). With three APs, an ADP increase could more readily be observed (9 out of 12, *CF* = 81 Hz) and the CFs for two or three APs were statistically similar (Figure 3H, 67 vs. 81 Hz, Kruskal–Wallis with Dunn's *post-hoc* test, *p* > 0.05). Similarly, an increase in ADP in L5 neurons was induced more frequently with three APs (11 out of 12 recordings) compared to two APs (Figure 3H, 5 out of 12 recordings). The CF in L5 was comparable between conditions of two APs (mean 46 Hz) and three APs (Figure 3H, mean 57 Hz, *p* > 0.05). Further, the CF for two APs was statistically comparable between L2/3 and L5 (*p* > 0.05) but significantly lower in L5 compared to L2/3 for three APs (Figure 3H, Kruskal–Wallis with Dunn's *post-hoc* test, *p* < 0.05).

To confirm that the frequency-dependent increase in ADP amplitude reflects dendritic calcium electrogenesis (Larkum et al., 1999a; Perez-Garci et al., 2006; Potez and Larkum, 2008; Ledergerber and Larkum, 2010), the non-selective calcium channel blocker cadmium was added to the extracellular solution. Addition of cadmium completely blocked the frequency-dependent increase in ADP. More specifically, in 6 out of 6 neurons recorded in the presence of extracellular cadmium, no increase in ADP could be observed using three APs. This is significantly different from the recordings without cadmium, where an increase in ADP could be observed in 20 out of 24 recordings when inducing three APs (Figure 3I, data L2/3 and L5 pooled, Fisher's exact test, *p* < 0.001). Together, these results show that in PFC, bursts of APs generated at sufficiently high frequencies can induce a frequency-dependent increase in ADP, indicative of calcium electrogenesis. The frequency of somatic AP spiking required for these dendritic spikes is lower for L5 neurons compared to L2/3 neurons.

**Figure 4:**
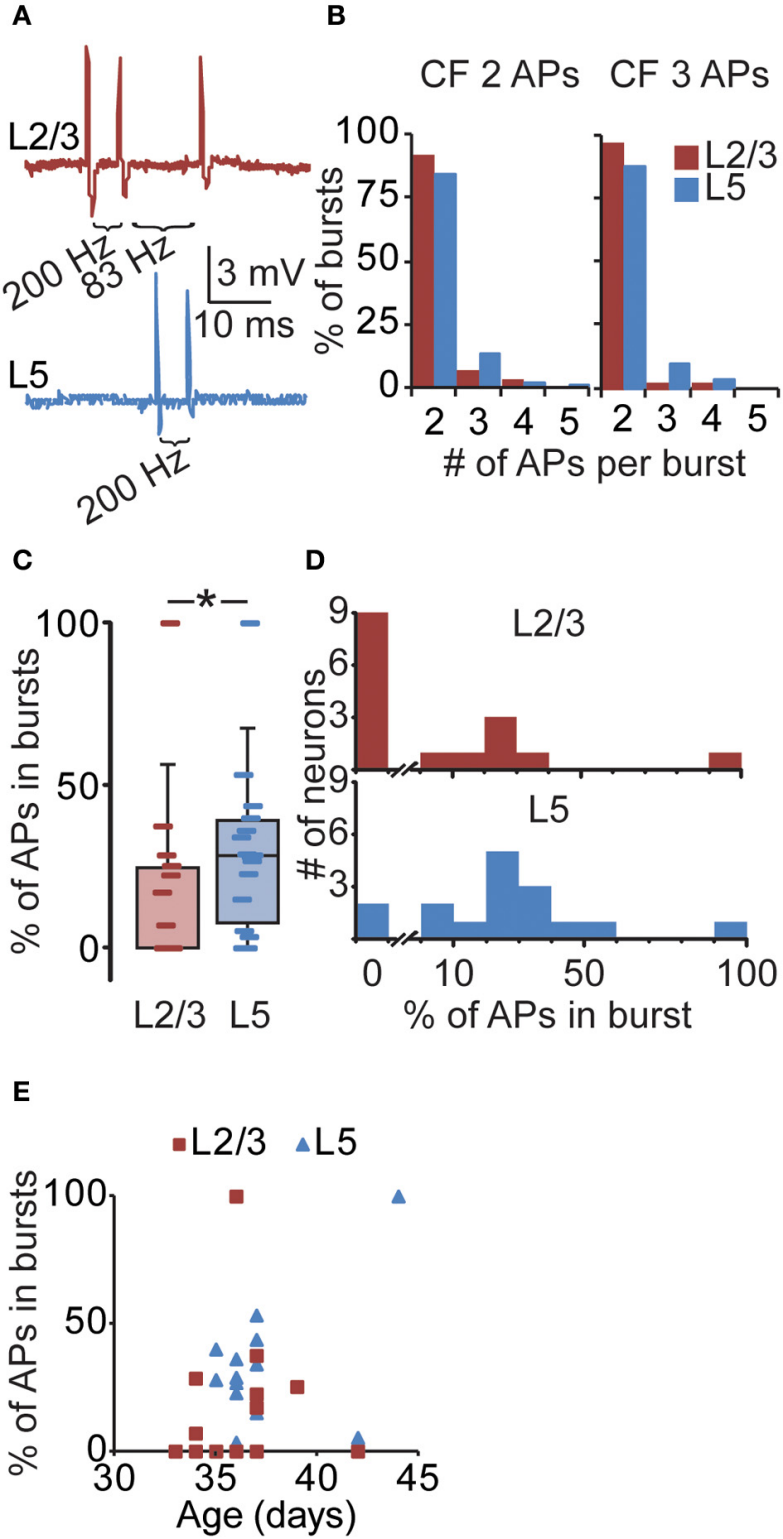
**Layer-specific likelihood of suprathreshold bursting in PFC. (A)** Upper panel: example of a L2/3 neuron illustrating three consecutive APs at 200 and 83 Hz. Lower panel: example of a L5 neuron illustrating two consecutive APs at 200 Hz. **(B)** The number of APs that are part of supracritical bursts for CF values obtained with two or three somatically evoked APs (see Figure 3). For example, the CF for L5 using two somatically evoked APs is 46 Hz (Figure 3). In our *in vivo* L5 recordings, we subsequently selected bursts exceeding the CF of 46 Hz and determined that 84% of such bursts consisted of two APs. Only a minority of supracritical bursts consisted of three, four, or even five APs at frequencies > 46 Hz. Note that the majority of supracritical bursts in L2/3 and L5 neurons thus contain two APs, both when using the CF for 2 spikes (L2/3: 67 Hz, L5: 46 Hz; left panel) and the CF for three spikes (L2/3: 81 Hz, L5: 57 Hz; right panel). **(C)** For individual PFC recordings from awake rats, we subsequently determined the percentage of APs that are part of supracritical bursts, defined as spiking frequencies exceeding the CF determined *in vitro* (Figure 3) and plotted these values in a box plot. **(D)** Same data as **(C)**, but plotted in a histogram (L2/3: upper panel, L5: lower panel), again illustrating the percentage of spikes in supracritical bursts for individual L2/3 and L5 neurons in awake rats. For instance, nine L2/3 neurons did not show any supracritical bursts compared to two L5 neurons. In general, the percentage of APs that were part of supracritical bursts was higher for L5 neurons compared to L2/3 neurons. ^*^denotes *p* < 0.05. **(E)** The relationship between the occurrence of bursts and age of the animal for L2/3 and L5 neurons.

Calcium electrogenesis in distal dendrites after high-frequency bursting has been shown to be comparable *in vitro* and *in vivo* (Waters et al., 2003). Therefore, to assess whether the layer-dependent increase in ADP amplitude *in vitro* is reflected in the likelihood for supracritical bursting *in vivo*, we next determined the occurrence of *in vivo* supracritical bursts using the *in vitro* CF measurements (Figure 4A). Using the CF for two APs (L2/3 67 Hz, L5 46 Hz, Figure 3) as threshold, we found that when instantaneous spiking frequencies exceed the threshold, 90.8% of supracritical APs were part of bursts consisting of two APs (Figure 4B). In L5, 84.0% of supracritical APs were part of two AP bursts. When we used the CF as determined with three APs *in vitro*, the supracritical bursts were also almost exclusively consisting of two APs (L2/3: 96.2%, L5: 87.0%). Thus, the majority of supracritical bursts in PFC of awake rats consisted of two APs. Therefore, we only used the CFs obtained with two APs *in vitro* to quantify the occurrence of bursts at CFs sufficient for calcium electrogenesis in awake animals. We found supracritical bursts in only 7 out of 16 (43.8%) L2/3 neurons (9 neurons with no supracritical bursts, median 0.00 (23.24)%). In contrast, both the proportion of L5 neurons that showed supracritical bursts was higher (14 out of 16 neurons, 87.5%, Fisher's exact test, *p* < 0.05), and also the population percentage of APs contributing to bursts was significantly higher compared to L2/3 neurons [Figures 4C,D, L5: 28.29 (24.53)%, Mann–Whitney U test, *p* < 0.05]. Taking only the neurons with supracritical bursts, the percentage of APs that are part of supracritical bursts is comparable [L2/3: 25.3 (13.3)%, *n* = 7; L5: 28.7 (15.2)%, *n* = 14, Mann–Whitney U test, *p* = 0.63]. Finally, it was reported previously that the age of the animal can have a significant effect on the probability of observing high-frequency bursting in L2/3 neurons of ferret visual cortex (Brumberg et al., 2000). We thus analyzed whether there is a correlation between bursting probability and age of the animal (Figure 4E), but for both L2/3 and L5 neurons, we did not find a correlation (Spearman correlation, L2/3 *p* = 0.21, L5 *p* = 0.50). This indicates that the occurrence of bursts in L2/3 or L5 is independent of the age of the animals used in this study.

In conclusion, we find sparse spiking in L2/3 and L5 neurons of PFC in awake rats, in addition to high-frequency bursts consisting of predominantly two APs. *In vitro*, these short bursts of two APs induced increases in ADP amplitude in a subpopulation of L2/3 and L5 neurons, indicative of dendritic calcium electrogenesis (Larkum et al., 1999a; Perez-Garci et al., 2006; Potez and Larkum, 2008; Ledergerber and Larkum, 2010). Finally, supracritical bursting occurs in a larger fraction of L5 neurons compared to L2/3 neurons indicative of cell-type-specific generation of dendritic electrogenesis. Thus, *in vivo* bursts exceeding frequencies for dendritic calcium electrogenesis *in vitro* suggests that dendritic electrogenesis may also occur in awake rats.

## Discussion

### Temporal dynamics of PFC activity

The sparse ongoing AP frequencies for PFC neurons are in close accordance with sparse spiking frequencies repeatedly documented for primary sensory areas (Barth and Poulet, 2012) and for single neuron recordings in PFC (e.g., Degenetais et al., 2002; Laviolette et al., 2005). In PFC, spiking frequencies in L2/3 and L5 were comparable for awake, isoflurane or urethane conditions. Comparing the cell-type-specific spiking, we found that the median spiking frequencies under awake conditions were comparable for L2/3 and L5 neurons but significantly higher in L5 compared to L2/3 under conditions of anesthesia (urethane or isoflurane), suggesting a cell-type-specific effect of urethane and isoflurane in PFC. Effects of urethane and isoflurane on cortical spiking or dendritic electrogenesis have been reported extensively (see, for instance, Erchova et al., 2002; Hara and Harris, 2002; de Kock and Sakmann, 2008; Potez and Larkum, 2008) but the cell-type-specific mechanistic basis of these anesthetics remains enigmatic. In the context of neuronal computation in PFC, however, we propose that awake conditions are most physiologically relevant for behavioral processing, and we therefore focused our efforts on temporal patterns of APs during awake conditions.

In the context of dendritic computation, instantaneous spiking frequencies are more informative than ongoing frequencies since the generation of dendritic calcium spikes depends on instantaneous frequency rather than ongoing activity. Irregular spiking and coincident bursts have been characterized in hippocampus and somatosensory cortex and consistently show that bursting properties and characteristics of dendritic computation are highly cell-type specific. For example, in primary somatosensory cortex, bursts are typically defined as two or three APs that occur at frequencies exceeding 100 Hz, and trains consisting of more than three APs are not frequently observed (de Kock and Sakmann, 2008). The CF for calcium electrogenesis is also cell-type specific and varies between 91 Hz for L5A pyramids (Grewe et al., 2010) and 130 Hz for L2/3 neurons (Larkum et al., 2007) with L5B neurons (100 Hz) and L6 neurons (96 Hz) exhibiting intermediate values (Larkum et al., 1999a; Ledergerber and Larkum, 2010). Here, we studied the increase in AP ADP amplitude after high-frequency AP bursting, which has been shown to be a reliable indicator of dendritic calcium electrogenesis (Larkum et al., 1999a; Perez-Garci et al., 2006; Ledergerber and Larkum, 2010). The average CFs at which an increase in the ADP amplitude could be observed are consistently lower in PFC (67 Hz L2/3, 46 Hz L5 using two APs), suggesting that passive and active properties of dendritic compartments of PFC neurons allow calcium electrogenesis at much lower spiking frequencies.

Previous studies have suggested that calcium dynamics in the PFC differ from those observed in somatosensory cortex (Seamans et al., 1997; Gulledge and Stuart, 2003). We analyzed the frequency-dependent increase in the AP ADP, and consistent with previous results (Larkum et al., 1999a), the increase in ADP after supracritical AP spiking in our experiments was completely abolished by the application of the non-selective calcium channel blocker cadmium, strongly suggesting that dendritic calcium electrogenesis occurs in PFC pyramidal neurons after high-frequency AP bursts. In addition, AP-induced calcium signals have been shown directly using calcium imaging in PFC neurons (Zhou et al., 2008), and in apical dendrites, frequency-dependent calcium electrogenesis could be observed with CFs in a similar range as those observed in the current study (Barth et al., 2008). Together, these results suggest that calcium electrogenesis in PFC can be induced after supracritical somatic AP spiking. Backpropagation of supracritical somatic AP bursts (as experimentally imposed during *in vitro* recordings) is one mechanism to induce calcium electrogenesis in distal dendrites (Larkum et al., 1999a), but the high-frequency bursts observed *in vivo* may actually reflect backpropagation-activated calcium spike (BAC) firing, which involves interaction between backpropagated APs and synaptic input (Larkum et al., 1999b; Murayama et al., 2009). Future experiments involving dendritic recordings or calcium imaging will be necessary to further characterize these events and the functional implications of dendritic electrogenesis in PFC.

We found that in awake rats high-frequency bursting occurs with similar likelihood in L5 of the PFC compared to L5B neurons of primary somatosensory cortex (de Kock and Sakmann, 2008). Furthermore, bursts in PFC typically consist of two APs (90%) and high-frequency trains of three or more APs are rare events, which is also consistent with sensory cortex L5B neurons. Our *in vitro* studies show that two APs are sufficient to induce dendritic electrogenesis in PFC neurons, although in a subpopulation of neurons three APs are required. Considering the temporal dynamics of ongoing AP spiking and bursts, our findings suggest highly conserved electrophysiological properties for L5 neurons across two functionally distinct cortical areas (de Kock and Sakmann, 2008). However, it is important to note that calcium electrogenesis could not be induced in a considerable proportion of neurons recorded *in vitro* in particular with two APs, leaving the possibility that also *in vivo* calcium electrogenesis may not always be induced with two suprathreshold APs. In addition, the slicing procedure severely changes the cortical network from its *in vivo* condition, although it should be noted that previous studies have shown that calcium electrogenesis induced by high-frequency bursts is a very robust phenomenon with similar properties *in vitro* and *in vivo* (Helmchen et al., 1999; Waters et al., 2003) and persists in the presence of increased synaptic input *in vitro* (Bar-Yehuda et al., 2008). Therefore, even though we did not directly record the presence of calcium electrogenesis *in vivo*, our data shows that ongoing spiking frequencies in awake animals regularly exceed CFs *in vitro* and suggests that calcium electrogenesis may occur *in vivo*.

Within cortical L5, two subtypes of pyramidal neurons can be found that show distinct morphological and physiological properties both *in vitro* and *in vivo* (Morishima and Kawaguchi, 2006; Dembrow et al., 2010; Oberlaender et al., 2011). We did not observe subgroups within L5 neurons on the basis of electrophysiological data or morphological properties without knowledge of projection target, which was used in previous studies to differentiate L5 neurons (Morishima and Kawaguchi, 2006; Dembrow et al., 2010). In addition, due to the technical challenge of filling neurons with biocytin in awake animals, the quality of the histological staining often did not permit the reconstruction of the full dendritic morphology. Therefore, we could not determine whether supracritical bursting correlates with morphological parameters on the level of individual neurons. Future studies should reveal whether L5 neurons in the PFC show subtype-dependent CF for calcium electrogenesis and *in vivo* high-frequency bursting.

### Functional implications of high-frequency bursting

The physiological and behavioral implications of bursts in PFC L5 neurons remain speculative, but for L5B thick tufted neurons of primary somatosensory cortex, a function was recently revealed (Xu et al., 2012). During a whisker-based object location task in awake mice, thalamic sensory input arriving at basal dendrites of L5B pyramids evokes somatic APs. These somatic APs backpropagate into the apical dendrite and amplify coincident synaptic input in the apical tuft from indirect (Mao et al., 2011; Oberlaender et al., 2011) or direct motor pathways (Petreanu et al., 2009; Xu et al., 2012). This coincident detection of sensory and motor signals results in dendritic calcium signals (Xu et al., 2012), which may induce a switch from single AP spiking in L5B neurons to burst spiking and hence increased output of L5B thick tufted neurons (Larkum et al., 2004; Oberlaender et al., 2011; Xu et al., 2012; Larkum, 2013). This increased AP output could influence sensory-guided behavior and dendritic spikes could thus directly contribute to cortical computation during sensation.

Whether similar mechanisms of coincidence detection exist in L5 neurons of PFC remains to be determined. PFC neurons are involved in integration of information from multiple projection pathways, including the midline thalamus, contralateral PFC, amygdala, and hippocampus (Hoover and Vertes, 2007) carrying distinct functional signals related to attention, emotion, and memory (Vertes, 2006). Similar to other cortical areas, anatomical inputs to the PFC are segregated and innervate different subcellular compartments of PFC neurons (Hoover and Vertes, 2007; Hirai et al., 2012; Little and Carter, 2012). Dendritic calcium electrogenesis, whether caused by high-frequency AP spiking at the soma or association of backpropagating APs with synaptic events in distal dendrites, could be a mechanism through which functionally segregated signals are integrated in the PFC, particularly in L5 neurons.

Thus, we showed that supracritical bursts frequently occur in L5 neurons and in a smaller subset of L2/3 neurons. It will be challenging but important to determine whether the occurrence of bursting is behavior-dependent. For instance, PFC neurons show significant changes in spiking properties and may even switch from single AP spiking to burst spiking in relation to a cognitive task (Burgos-Robles et al., 2007; Totah et al., 2009). This increased bursting could reflect integration of functional signals related to cognitive processing and is critical for correct decision making. To understand the computational power of individual PFC neurons during cognitive performance and the interplay between segregated information streams, it will be important in future studies to reveal the subcellular organization of the different input pathways and to determine how coincident activity of segregated pathways influences PFC output and cognitive behavior.

## Author contributions

Zimbo S. R. M. Boudewijns, Rhiannon M. Meredith, Huibert D. Mansvelder and Christiaan P. J. de Kock designed research; Zimbo S. R. M. Boudewijns performed and analyzed *in vivo* recordings; Martine R. Groen performed and analyzed *in vitro* recordings; Brendan Lodder reconstructed neurons; Minni T. B. McMaster and Lawrence Kalogreades assisted with *in vivo* recordings; Rajeevan T. Narayanan assisted with histology; and all authors were involved in writing the paper.

### Conflict of interest statement

The authors declare that the research was conducted in the absence of any commercial or financial relationships that could be construed as a potential conflict of interest.
